# When running is easier than walking: effects of experience and gait on human obstacle traversal in virtual reality

**DOI:** 10.1007/s00221-022-06443-2

**Published:** 2022-09-17

**Authors:** Florian Hofmann, Volker Dürr

**Affiliations:** 1 Biological Cybernetics, Faculty of Biology, Bielefeld University, Universitätsstr. 25, 33615 Bielefeld, Germany; 2 Centre for Cognitive Interaction Technology, Bielefeld University, Bielefeld, Germany

**Keywords:** Kinematics, Obstacle, Human locomotion, Gait

## Abstract

Humans readily traverse obstacles irrespective of whether they walk or run, despite strong differences between these gaits. Assuming that the control of human obstacle traversal may be either gait-specific or gait-independent, the present study investigates whether previous experience in an obstacle traversal task transfers between the two gaits, and, if this was the case, whether transfer worked both ways. To this end, we conducted a within-group comparison of kinematic adjustments during human obstacle traversal in both walking and running, with distinct participant groups for the two gait sequences. Participants (*n* = 12/12 (f/m), avg. 25 yo) were motion captured as they traversed obstacles at walking and running speeds on a treadmill, surrounded by an immersive virtual reality (VR) environment. We find that kinematics recorded in our VR setup are consistent with that obtained in real-world experiments. Comparison of learning curves reveals that participants are able to utilize previous experience and transfer learned adjustments from one gait to another. However, this transfer is not symmetrical, with previous experience during running leading to increased success rate in walking, but not the other way round. From a range of step parameters we identified lacking toe height of the trailing leg as the main cause for this asymmetry.

**Supplementary Information:**

The online version contains supplementary material available at 10.1007/s00221-022-06443-2.

## Introduction

Being able to react to obstacles is fundamental for safe and stable locomotion in everyday environments. Not unlike animals, humans frequently step across obstacles, be it loose items on an apartment floor, puddles or stones on a sidewalk or tree roots on a jogging trail in the woods. In general, we are able to adjust locomotor parameters quickly and appropriately for a wide range of obstacle sizes and irrespective of gait (e.g., walking vs. running).

With regard to motor control it is not trivial to traverse an obstacle in distinct gaits. Traditionally, the bipedal human gaits walking and running have been regarded as being fundamentally different in terms of biomechanics, with abrupt changes between the gaits (Alexander 2013). As a consequence, one might expect gait-specific adjustment in the control of obstacle traversal.

An alternative view suggests that walking and running may be considered as two parametrisations of the same underlying control system (Geyer et al. 2006) linked to a common underlying mechanical system (Hildebrand 2013). Physiological evidence from cats shows that increasing stimulation strength of the mesencephalic locomotor region (MLR) evokes locomotion with increasing speed, including appropriate gait changes (Mori et al. 1989). In humans, EMG recordings of walking and running participants show remarkable similarities in the temporal activation patterns across gaits (Cappellini et al. 2006). Finally, toe trajectories are similar over a wide range of conditions and disturbances (e.g., Lam and Dietz 2004), suggesting that control happens spatially for the end effectors, while timing and magnitude of muscle activation are adjusted accordingly. In the light of this background, the present paper focuses on the spatial differences between the gaits. Obstacle traversal may be achieved for both gaits in the same way by gathering spatial information on obstacles via visual, proprioceptive or auditory cues and adjusting parameters (e.g., necessary toe height) of a central control system in only one gait. The mechanism underlying transfer of the learned adjustments would then be implicit, with timing and magnitude of muscle activity adjusted to achieve different speeds in either gait, with the resulting spatial end effector trajectories remaining the same.

To differentiate between the alternatives of gait-specific or gait-independent adjustments, we test to what extent successful obstacle traversal in one gait provides sufficient experience for successful traversals in the other gait. As motivated above, we do so by analysing the spatial adjustments to trajectories over obstacles and success rates of human obstacle traversals in walking and running within the same group of participants.

Traditionally, research on obstacle traversal has treated gaits separately: In walking, trajectories of the leading foot are not adjusted during ongoing steps. Rather, adjustments are planned ahead during the last steps of the approach, based on visual judgement of obstacle size and distance (Patla et al. 1996, 1991; Patla and Greig 2006; Patla and Rietdyk 1993; Patla and Vickers 1997). Trajectories of both feet are scaled according to obstacle height in both real, physical environments (Patla and Rietdyk 1993) and Virtual Reality (VR; Binaee and Diaz 2019). This applies even if obstacles are traversed “from memory”, i.e., without visual control (Heijnen et al. 2014).

Other than for human walking, much less is known about obstacle traversal during human running (Mauroy et al. 2013), an exception being highly trained obstacle crossing in athletics hurdling races. Alexander (1984) argued that the spring-like properties of the leg were additionally loaded by lowering the Center of Mass (COM) before crossing obstacles. The combination of stored energy in the leg-spring and the lever of the leg-spring then releases sufficient potential energy to increase the COM height over the obstacle (Alexander 1984; Mauroy et al. 2013).

Compared to the guided trajectories of the leading foot, the shape of these evasive trajectories of the trailing foot is more variable (Patla et al. 1996), their height being planned independently of the leading foot movement (Patla et al. 1996) or derived from the leading foot (Sparrow et al. 1996).

Irrespective of the mechanism that is responsible for planning trailing foot trajectories, two factors can lead to collisions. First, improper foot placement before the obstacle will displace the intended trajectory relative to the obstacle, increasing the chance of collision, even though sufficient height was achieved (Chou and Draganich 1998). Second, if foot placement was adequate, insufficient height might still be a factor leading to collision (Heijnen et al. 2012). A distinction must be drawn here between young and older adults (< 35 and > 65 years) with the former showing adequate step positioning with collisions happening more often with the trailing leg, whereas the latter show improper foot placement with more collisions of the leading foot (Muir et al. 2020).

To tell gait-dependent from gait-independent adjustments to previously un-encountered obstacles, our study was designed to show short-term adaptation to traversals over obstacles in a VR environment. Earlier VR studies on human obstacle traversal found the behaviour recorded in VR to be similar to real-world behaviour (Binaee and Diaz 2019), provided that the obstacle is presented from a first-person point of view [when presented from a third-person perspective, success rates are lower, e.g., see LoJacono et al. (2018)]. In our case, we exploit the learning curve as participants traverse virtual obstacles and contrast overall success rates and kinematic parameters in paired walking and running trials. To the best of our knowledge, this is the first within-group comparison of kinematic adjustments during obstacle traversals in running and walking participants. As a consequence, our data allows us to distinguish whether an effect is specific to the current gait or rather a result from a general, gait-independent strategy for adjustment of human locomotion. Moreover, it allows us to monitor the change of success rates over time for both gaits and, therefore, address the transfer of learned adjustments between gaits.

Our main hypotheses were that (i) trained kinematic adjustments during obstacle traversal transfer from one gait to another, and (ii) that this transfer is symmetrical, i.e., works similar irrespective of the gait that was used during training. This would be expected if the execution of gaits is subject to spatial modulation that is independent of the ongoing motor pattern. Alternatively, if kinematic adjustments did not transfer across gaits at all, spatial adjustments would have to be gait-specific. A third possibility would be that transfer would work asymmetrically, i.e., would depend on which gait was used during training.

Our results show that experience with successful traversals does transfer across gaits, but not in a symmetrical manner: successful traversal during running transfers to walking but not the other way round.

## Materials and methods

### The interactive locomotion lab

**Fig. 1:**
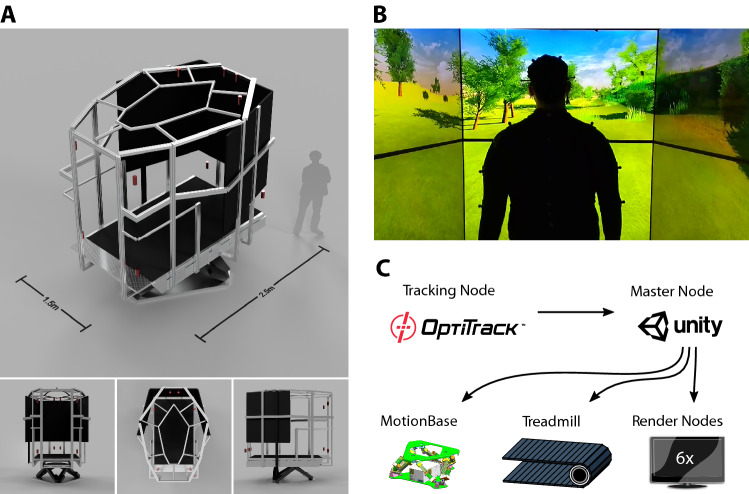
Interactive Locomotion Lab (ILL). **A** The ILL is composed of a Stewart motion platform that carries a treadmill, 6 TV screens and a motion capture system with 14 cameras (approximate positions in red). Three lower panels show the front, top and left side view of the ILL. **B** Participant view of the visual environment. **C** System components of the ILL. Full body tracking data is recorded on a dedicated computer (Tracking Node) and incorporated into the Unity scene that is computed on the Master Node. The latter also controls the position and orientation of the motion platform and treadmill speed. The visual scene is rendered by three dedicated Render Nodes and displayed via 2 × 3 TV screens

To facilitate comparative locomotion studies, we set up an “Interactive Locomotion Lab” (ILL) with a combination of hardware and software systems developed for studying human and robot locomotion (Fig. 1). It comprises a treadmill mounted on a motion base with six degrees of freedom (DOF), a Virtual Reality (VR) setup in the form of six screens arranged in a half hexagon, and a motion capture system.

### Motion base

The motion base comprised a 2.5 × 1.5 m^2^ treadmill (Achilles, Rheda-Wiedenbrück) mounted to a Stewart motion platform with six DOF (Moog 170-140B-2-D-1; Moog Germany GmbH). The treadmill area was 2.5 × 1.5 m, allowing stationary running of a tall human even at high speeds without getting too close to the treadmill borders. An aluminium frame was mounted to the motion base to support the screens of the VR system and motion capture cameras (Fig. 1A). As the cage was not perfectly rigid, some accuracy was lost due to warping under movement. Tracking accuracy during operation of the motion platform was measured to be 0.39 ± 0.30 mm.

### Virtual reality setup

The VR system comprised six 65" flat-screen televisions (Sony Bravia X85) with a maximum resolution of 3840 × 2160 at 60 Hz, and a maximum viewing angle of 89°. The upper monitors were mounted upside down to reduce the inter-screen gap to 2 cm. The entire system was controlled from a central program utilizing the Unity^®^ 2017.4.0f1 game engine (Fig. 1C, Unity Technology, San Francisco, U.S.A.). UniCave (Tredinnick et al. 2017) was used for head tracking of the participants and distributed rendering of the VR scene.

**Fig. 2:**
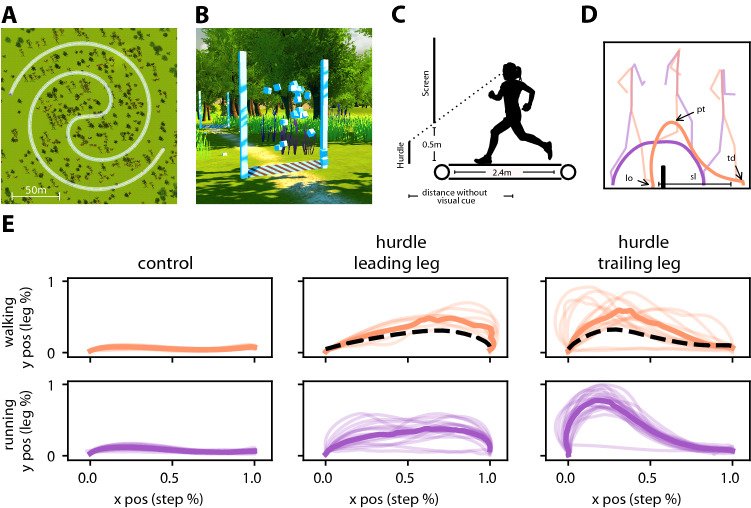
Virtual track, hurdles and single-step analysis. **A** Top view of the virtual scenery. The running track was a Fermat spiral with a track length of 729 m. **B** Hurdles were visually distinct from the environment. Hurdle width equalled treadmill width. Their height ranged between 0.1 and 0.4 m. To the left and right of the hurdle, two columns were displayed that did not leave the field of view before crossing the hurdle, thus indicating the position relative to the hurdle. **C** Low visual features left the field of view shortly before they were passed. **D** Sample trajectories of the leading (violet) and trailing (orange) foot, with a hurdle in black. Key parameters are indicated for the trailing foot. Pale lines show three full-body postures during the traversal. **E** Toe trajectories are similar during walking and running, and for real and virtual hurdles. Left **c**ontrol trajectories of steps without hurdle. Middle: toe trajectory of the leading foot traversing a hurdle. Right: toe trajectory of the trailing foot traversing the hurdle. Bottom row: walking (orange); top row: running (violet) each. Thin lines show median trajectories per participant; thick lines show the median over all participants. For comparison, black dotted lines show mean trajectories from (Patla and Rietdyk 1993) (hurdle height = 20 cm). Note that one participant was unable to complete the task successfully

The monitors were arranged in a semi-hexagon with two rows and three columns (see Fig. 1A). The central column was aligned with the running track, while the left and right columns were rotated by 60°. From a viewing position 170 cm above the centre of the running track, the VR screen subtended a vertical viewing angle of 64° and a horizontal viewing angle of 180° (Fig. 1B). Since the lower rim of the screens was 0.5 m above the treadmill surface, ground features left the field of view before passing the participant (see Fig. 2C). Assuming an eye height of 1.7 m above the treadmill centre, this happened at a distance of 1.62 m (0.37 m behind the screen).

### Motion capture

Motion data was captured with an Optitrack system (Natural Point Inc., Corvallis/OR, U.S.A.; Optitrack Motive 1.9) consisting of 12 Flex* 13* cameras mounted at various locations on the support cage (red symbols in Fig. 1A). After calibration, spatial accuracy of marker tracking was approximately 0.2 mm at a temporal sampling frequency of 120 frames per second (fps).

Participants wore an Optitrack full-body motion suit with 52 markers in the “Biomech configuration” as specified in (https://v21.wiki.optitrack.com/index.php?title=Biomech_(57)) and 6 markers on glasses to track the head position. 6 DOF data for 21 skeleton segments was recorded at 120 fps, independent of the frame rate of the Unity VR system. The head tracking data was used to adapt the projection to the participant’s head position. In addition, the position, orientation and pose of the participant were captured in the global coordinate system of the VR world, allowing the reconstruction of the full skeleton model in the VR environment. The motion-to-photon latency (Yao et al. 2014) between the motion-capture and VR display was experimentally measured to be 120 ms.

### Safety measures

To mitigate the risk of injury even at running speeds up to 4 m/s, an emergency stop system was put in place that employed mechanical brakes to stop both the treadmill and any movement of the platform in less than 10 ms. To prevent injury in case of falling, participants wore a full-body safety harness fixed to the ceiling with a retracting lifeline. The harness allowed natural movement without restricting the participant.

### Experiment 1

The main purpose of experiment 1 (exp. 1) was to show that human obstacle negotiation on our VR setup is consistent with published data acquired in real-world environments. As it also revealed differences between gaits that gave rise to the subsequent experiment 2 (exp. 2) on symmetrical transfer among gaits, a second purpose of exp. 1 is to show replicability of the observed learning curve in walking. The virtual scenery of exp. 1 was an open woodland with rolling hills (Fig. 1B, 2B). The participants were guided along a trail marked by white stones on the ground. The width of the virtual path corresponded to the width of the treadmill, i.e., 1.5 m. Scene assets were created with the free Nature Starter Kit 2 (Shapes, Unity Asset Store). The treadmill speed corresponded to the simulated visual motion.

The running track was designed in the shape of a Fermat spiral (Fig. 2A). Total track length was 729 m. Only the last quarter of the track contained 18 hurdles (Fig. 2B) which were spaced evenly with 10 m intervals. To exclude that participants could adjust their foot height to a particular obstacle size (see Lam and Dietz 2004), hurdle heights were sampled at random from a uniform distribution ranging from 0.1 to 0.2 m, corresponding to 9–18% of the leg length of the average participant. The height sample was the same for all participants and across gaits. Hurdle widths equalled the treadmill width. Hurdle depth was set to 0.05 m. Two poles of 2 m height were placed on either side of each hurdle to indicate the position of the hurdle after it had left the field of view (Fig. 2B, C). This was found to improve step positioning when vision was obstructed (Rietdyk and Rhea 2006).

In total, 23 adult participants (9 female, 14 male, age: 25.04 ± 4.51) took part in the first experiment. Each participant completed the trail twice, where the first trial was at walking speed (1 m/s) and the second trial was at slow running speed (2 m/s). The same 18 hurdles were encountered during both trials. As both legs were tracked, each participant contributed 72 hurdle trajectories. Audible feedback was given for each obstacle, indicating whether the traversal was rated as a collision or a successful traversal. Collisions were determined by a simple collider model in Unity, using two spheres of 0.05 m diameter around the toe and the foot joint positions reported by Optitrack. As improper foot placement with one foot may result in failures with the other foot, collisions were evaluated “per hurdle”. Even if only one foot did collide, both trajectories that crossed that hurdle, i.e., from both feet, were labelled as collisions.

Participants were instructed which gait to use and to cross the hurdles as naturally as possible. If they struggled with the task, advice was given about the lift-off point (too early, too late), height of the jump (too low, too high). Most participants were able to perform the task successfully, with 20 of the 26 participants successfully clearing more than 50% of the hurdles and 68 ± 17% of all hurdles cleared successfully.

### Experiment 2

24 adult participants (12 female, 12 male, age: 24.92 ± 6.40) took part in the second experiment. It was conducted according to the same protocol as exp. 1, except for changes in trial order, hurdle height, distance and number. The order of the walking and running task was swapped for every second participant resulting in two cohorts W and R, corresponding to the gait that was used in the respective first trial. The number of hurdles was increased to 36 per gait, and the distance between hurdles was reduced to 5 m. The maximal height of the hurdles was increased to 0.4 m, with a single set of heights drawn from the uniform distribution between 0.1 and 0.4 m shared across participants and gaits. Again, most participants were able to perform the task successfully, with 19 of the 24 participants successfully clearing more than 50% of the hurdles and 71 ± 24% of all hurdles cleared successfully.

Both experiments were approved by the University’s Human Research Ethics Committee. All participants gave their informed written consent, and filled out a questionnaire about their eyesight, health condition.

### Data analysis

Raw data from the Optitrack system was transformed to and aligned temporally with the corresponding VR data sets: data recorded within the local frame of reference of the motion capturing system was “unrolled” into the global coordinate system of the VR system by cumulating the constant distance covered per sample.

21 skeletal joint positions were estimated by the motion capture system. Of these, only the toe trajectories were taken from the Toe joint of the foot segment, corresponding to the center of the metatarsophalangeal joints. COM position was assumed to coincide with the location of the Hip joint in the center between the two acetabuli.

Stance phases were defined as episodes in which the vertical coordinate of the toe marker was below 0.05 m. From there, lift-off positions and resulting step lengths were established from the end of a stance phase to the beginning of the next. Individual trajectories over hurdles were extracted by discarding all step trajectories that did not start before and end after a hurdle position. Trajectories containing more than 10 untracked values were discarded, too, leaving one participant without valid trajectories. Of the 1976 trajectories that were recorded in the first experiment, 746 trajectories were excluded due to tracking errors. Of the 3848 trajectories that were recorded in the second experiment, 1606 trajectories were excluded due to tracking errors.

Six parameters were extracted for each valid trajectory and for both feet: peak COM height, COM crossing height, peak toe height, toe crossing height, toe clearance, lift-off and touch down distances and step length. Thus, a total of twelve parameters were obtained per obstacle. For the COM and toe markers, peak height was defined as the highest point of the trajectory of the marker and the height at crossing was sampled vertically above the hurdle in the instance of passing. Toe clearance was defined as the minimal distance of the toe marker to the hurdle at the instance of passing. Lift-off and touch-down distances were defined as horizontal distances relative to the hurdle, as the points, where the toe marker crossed a height of 0.05 m, initiating a swing trajectory. Finally, step length was defined by the difference between touch-down and lift-off distances.

### Statistical analysis and modelling

The statistics module of the Python library *scipy* version 1.3.1 was used for statistical analysis without any special parameter set. Nonlinear models were fitted using the Python libraries *numpy* 1.16 and *scipy* 1.3.1. Statistics were calculated on independent measures as data points were grouped and averaged per participant. Paired tests were calculated at the expense of reduced sample sizes whenever some participants did not contribute to both samples. This could happen, if participants never collided with any obstacle in a certain task, thus not contributing data points to an “unsuccessful traversal” sample. Boxes of boxplots show medians, 1st and 3rd quartiles, while whiskers show the minimum and maximum values, with outliers marked individually whenever exceeding 1.5 times the interquartile range beyond the box.

Ordinary least square models were used to estimate the initial performance (intercept) and increase in performance (slope) for all trials. To evaluate the effect of the 2 × 6 trajectory parameters on participant success rates, multiple non-linear mixed effect models were fitted to the data set. In a first step, models were tested systematically by introducing one factor at a time and testing whether their introduction significantly reduced the residual error (*ɛ*). Out of the factors described above, only three proved to have a statistically significant effect on performance: these were toe height, lift-off distance and step length. In no iteration, any of the other factors significantly increased the residual error. Accordingly, Eq. 1 was chosen as the model to predict success rates per hurdle and participant:

1 $$success\sim \left({\beta }_{1}+{\beta }_{P}\right)\cdot E\left(fh,{\beta }_{fm},{\beta }_{fv}\right)\cdot G\left(lo,{\beta }_{lm},{\beta }_{lv}\right)\cdot E\left(sl,{\beta }_{sm},{\beta }_{sv}\right)+\upvarepsilon $$

where $gauss\left(x,b,c\right)={e}^{\frac{-{\left(x-b\right)}^{2}}{2{c}^{2}}}$ and $expit\left(x,b,c\right)=\frac{1}{1+{e}^{\left(b-x\right)\cdot c}}$ are Gaussian and Binomial inverse link functions, respectively.

In Eq. 1*β*_*1*_ is the average success rate across participants, and *β*_*p*_ is a random effect per participant. *β*_*fm*_ and *β*_*fv*_ are the mean and slope of the inverse link function for parameter toe height *fh*; *β*_*lm*_ and *β*_*lv*_ are the mean and standard deviation of the inverse link function for parameter lift-off distance *lo*; and *β*_*sm*_ and *β*_*sv*_ are the mean and slope of the of the inverse link function for parameter step length *sl*.

In a second step, models including the three remaining factors (*success*_*factor*_ in Eq. 2) were fitted and compared to a null model, success_0_:

$${success}_{0}\sim \left({\beta }_{1}+{\beta }_{G1}+{\beta }_{P}\right)$$

2 $${success}_{factor}\sim \left({\beta }_{1}+{\beta }_{G2}+{\beta }_{P}\right)\cdot {p}_{factor}$$

where β_1_ and β_P_ and p_factor_ correspond to mean success rates and the factor terms of Eq. 1, respectively. *β*_*G1*_ and *β*_*G2*_ were introduced to capture the difference in success rate between the two groups (i.e., trial sequences in exp. 2).

Models were bootstrapped by case-sampling (*n* = 1000) to obtain error bounds. Whether or not a given parameter explained a significant difference between the two cohorts was tested with *t* tests on *β*_*G1*_ and *β*_*G2*_.

## Results

### Success rates of obstacle crossing in VR improve over time

To establish the validity of our virtual obstacle crossing paradigm, we first made sure that participants performed naturally and successful under VR conditions in the Interactive Locomotion Lab (ILL).

To this end, we conducted exp. 1, in which participants first walked and then ran along a path in a virtual environment (Fig. 1B, 2). During both trials, participants encountered the same set of 18 virtual hurdles. All but one participants crossed the virtual hurdles with naturally looking foot trajectories (see qualitative comparison with literature data in Fig. 2E) suggesting that the overall movement observed in the ILL was equivalent to that in a physical environment. Only one participant did not manage to perform the task even at the end of both trials. Their data points were still included, trusting that this will only increase the number of outliers.

**Fig. 3:**
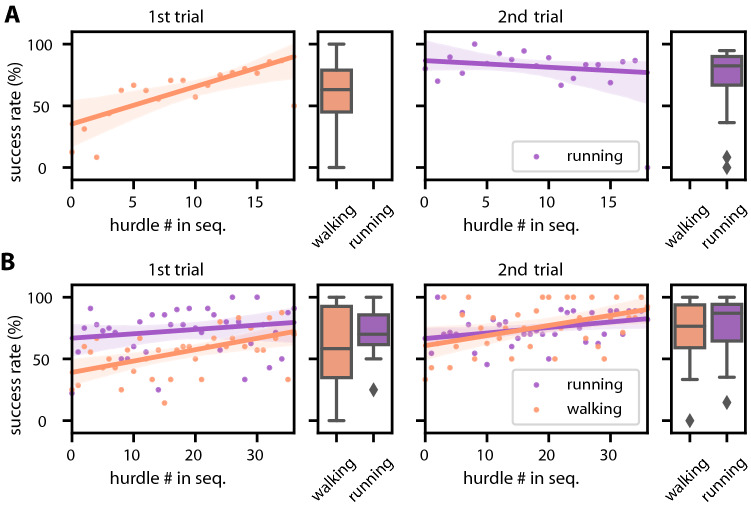
Success rates per hurdle in experiments 1 and 2. **A** In exp. 1 hurdle ID corresponds to number in sequence. Hurdle heights were randomised once but the same sequence was used for all participants (*N* = 26). During walking (trial 1), success rate increased continuously with number of traversed hurdle. During running (trial 2) success rates remained constant. Solid lines and shaded areas show linear regression with 95% confidence bounds. Dots show success rate averages per hurdle; box plots show mean success rates per participant. **B** In exp. 2 hurdle ID corresponds to number in sequence. All participants experienced the same random sequence of hurdle heights. Cohort W (*N* = 12) first walked, then ran, whereas cohort R (*N* = 12) first ran, then walked. As in exp. 1, success rates increased during walking, irrespective of whether or not participants had traversed hurdles before. During running trials, success rates increased less than during walking. Solid lines and shaded areas show linear regression with 95% confidence bounds. Boxplots show medians, 1st and 3rd quartiles, while whiskers show the minimum and maximum values, with outliers marked individually whenever exceeding 1.5 times the interquartile range beyond the box

Moreover, 20 participants crossed at least half of the hurdles successfully. Overall, 68% of all hurdles were cleared successfully (Fig. 3A), though with a significant difference in success rates between the initial walking trial (59%) and the subsequent running trial (77%). Typically, participants started out below the average success rate, but improved their performance significantly throughout the walking trial (Spearman: *M* = 0.918, *p* < 0.001). During the running trial, their performance stayed constant (Spearman: *M* =  −  0.122, *p* = 0.630). The continuous improvement in trial 1 suggested that participants learned how to cross the hurdles successfully. As the trial order was the same for all participants, we could not tell whether the constant performance during running was due to successful transfer of learning from walking to running, or rather due to immediate successful traversal, i.e., due to the task of hurdle crossing being easier during running than during walking.

### Transfer across gaits is not symmetrical

To distinguish between these two alternatives, we conducted a second experiment in which the initial gait was alternated, such that half of the 24 participants started out with walking (cohort W), the other half with running (cohort R). Despite the larger range of hurdle heights in exp. 2, average performance was similar to that in exp. 1, and 70% of all hurdles were cleared successfully (neither foot collided). The overall success rates for walking was 64% vs. 75% in the running task (Fig. 3B).

In the walking trials, the initial success rates (intercepts of linear models in Fig. 3B) were 39 ± 32% vs. 61 ± 37% for cohorts W and R, respectively (*t* Test: *t* = − 2.680, *p* < 0.01). In the running trials, the initial success rates were 66 ± 23% vs. 66 ± 35% (*t* = 0.042, *p* = 0.966) for cohorts W and R, respectively. In the walking trials, the increase in performance over the course of the trial was 33 ± 09 percent points (Spearman: *M* = 0.513, *p* < 0.01) vs. 29 ± 10 percent points (*M* = 0.359, *p* < 0.05) for cohorts W and R, respectively (*t* = 6.649, *p* < 0.01). In the running trials, the increase in performance was 16 ± 06 percent points (*M* = 0.340, *p* < 0.05) vs. 12 ± 10 percent points (*M* = 0.165, *p* = 0.335; *t* = 1.460, *p* = 0.158). These numbers are reported “per-hurdle” as described above. When considering the two limbs individually 25% and 20% of all collisions during walking occurred in the leading and trailing foot, respectively, compared to 19% and 17% during running. In summary, previous experience in the running trials increased the initial success rate in subsequent walking trials, whereas initial success rate and increase in performance in the running trials were independent from previous trials. We conclude that motor improvement does transfer from one gait to another, however, only from *running to walking*.

### Differences between cohorts do not contribute to differences in success rates

**Fig. 4:**
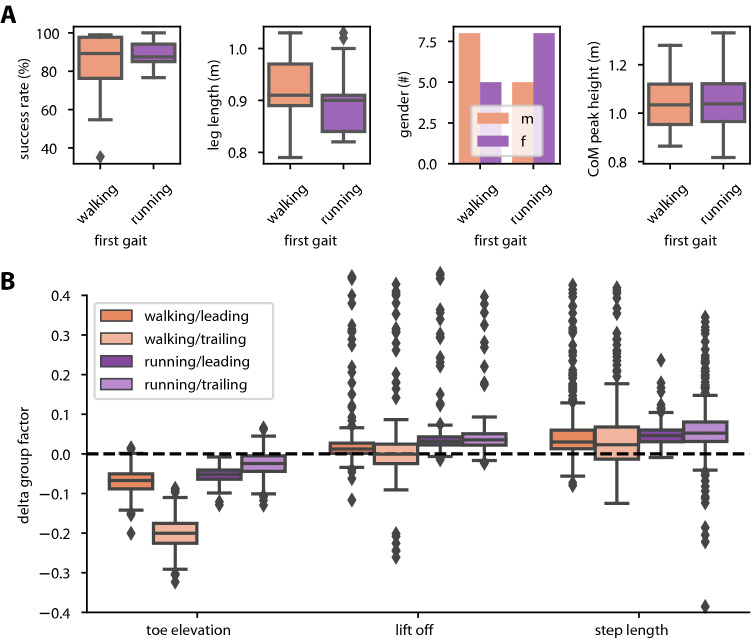
Only peak toe height differs strongly between cohorts. **A** The gap in success rates between the walking trials of the cohorts cannot be explained by the factors leg length, gender, hurdle heights, COM height or an overall difference in success rate. **B** Peak toe height differs most strongly between cohorts. Lift-off distance and step length have much weaker effects. Box plots show inter-model group factor differences (Δβ_12_ in Table 2). Differences were obtained by comparing the group factors between full and null models. Peak toe height of the trailing foot differs by 20% between the two groups, while the next largest difference is less than 7%. All factors differ significantly from zero, but have too little magnitude as to explain a difference between cohorts. 75% of outliers in the plots of parameters lift-off and step length have been cropped for clarity. Boxplots show medians, 1st and 3rd quartiles, while whiskers show the minimum and maximum values, with outliers marked individually whenever exceeding 1.5 times the interquartile range beyond the box

To make sure that this asymmetry was really an effect of different motor behaviour and not induced by other differences between the cohorts we tested for inter-group differences in leg length, group gender composition and overall success rates (Fig. 4A). Noteworthy, the correlation between leg length and mean success rate was weak and non-significant in both walking (Spearman: *M* = 0.379, *p* = 0.075) and running (Spearman: *M* = 0.310, *p* = 0.140). More importantly, leg length did not differ significantly between cohorts (Fig. 4A, μ_1_ = 0.92 ± 0.07 m, μ_2_ = 0.90 ± 0.07 m; Mann–Whitney *U*: *U* = 70, *p* = 0.243) and, therefore, can be ruled out as a cause for the asymmetry. In addition, there was no gender bias between the cohorts (53% vs 33% female, *χ*^2 ^= 0.11, *p* = 0.733; Fig. 4A) that could have given rise to the asymmetry between cohorts.

As hurdle height varied between 0.1 and 0.4 m, its variation introduced an expected correlate of traversal success in both walking (Spearman: *M* =  −  0.367, *p* = 0.028) and running (Spearman: *M* =  −  0.733, *p* < 0.001). However, since both cohorts negotiated the same parkour, hurdle height can be ruled out as a cause of asymmetry. Finally, overall performance was not significantly different between the two cohorts (Fig. 4A, first from left, μ_walking_ = 83 ± 21%, μ_running_ = 89 ± 06%; Mann–Whitney *U*: *U* = 66, *p* = 0.386).

### Three foot trajectory parameters affect success rate

Having ruled out that differences between cohorts could have given rise to the asymmetry observed in exp. 2, we tended to movement-related parameters. Given that the biomechanical differences between the two gaits are related to leg compliance and, as a consequence, the trajectory of the ‘centre of mass’, COM, we first tested whether unsuccessful traversals could have been caused by gait-related differences in maximum COM height.

Peak COM height was sampled in a 5 m range around the hurdle. There was no difference in COM height between successful and colliding traversals in any of the trials (first running: μ_success_ = 1.07 ± 0.08 m, μ_collide_ = 1.06 ± 0.08 m, Wilcoxon’s *W* test for matched pairs: *n* = 11, *W* = 20, *p* = 0.248; first walking: μ_success_ = 0.95 ± 0.06 m, μ_collide_ = 0.94 ± 0.07 m, *W*: *n* = 7, *W* = 8, *p* = 0.310); second running: μ_success_ = 1.07 ± 0.08 m, μ_collide_ = 1.05 ± 0.06 m, *W*: *n* = 10, *W* = 11, *p* = 0.093; second walking: μ_success_ = 0.97 ± 0.09 m, μ_collide_ = 0.98 ± 0.10 m, *n* = 9, *W* = 18, *p* = 0.594).

Moreover, there was no significant difference between the COM height of successful traversals in the walking trials of the two cohorts (μ_1st_ = 0.97 ± 0.09 m, μ_2nd_ = 1.00 ± 0.10 m, Mann–Whitney *U*: *n*_1st _= 9, *n*_2nd_ = 12, *U* = 46, *p* = 0.297).

Therefore, we rule out insufficient COM height as the cause for collisions. Furthermore, we conclude that the improvement observed in Fig. 3A, B must have been related to changes in leg movement only.

Since our collision detection was based on foot trajectories, we tested for correlations between success rate and 6 foot trajectory parameters. Leading and trailing feet trajectories have not been considered seperately here. Only peak toe height, lift-off distance and step length were found to correlate with success rate (including random effects per participant). Ablating any of the identified parameters significantly increased the residual error of the model, proving that it was the most parsimonious model (Table 1).

**Table 1:** Effects of foot trajectory parameters and individual participants on traversal success

Full	Residuals:	0.192 ± 0.135
Full − toe height	ΔResiduals:	+ 0.093 ± 0.106: *p* ≪ 0.001
Full − participant random effects	ΔResiduals:	+ 0.033 ± 0.064: *p* ≪ 0.001
Full − lift-off distance	ΔResiduals:	+ 0.099 ± 0.088: *p* ≪ 0.001
Full − step length	ΔResiduals:	+ 0.042 ± 0.073: *p* ≪ 0.001

The full model is given by Eq. 1. Effects of single factor ablation were verified with Wilcoxon tests

Once identified, we tested the effect of these parameters on the inter-group difference Δβ_12_ by first estimating their cohort-specific differences, β_G2_, relative to the differences β_G1_ in a null model (see Eq. 2) and then comparing the bootstrapped distributions of β_G1_ and β_G2_ with pairwise *t* tests. The resulting pairwise differences (Δβ_12_ in Table 2) thus correspond to the effect of a given parameter on the difference in performance between cohorts. Since we wanted to interpret the statistical modelling results with regard to their effect on gait and the specific movement trajectories of the leading and trailing foot (see also Fig. 2D), we further subdivided trajectory samples into the four combinations of gait × foot (Table 2; Fig. 4B).

**Table 2:** Peak toe height differs most strongly between cohorts.

	β_G1_	β_G2_	Δβ_12_	*t*	*p*
Lift-off distance					
Walking/leading	+ 0.145 ± 0.037	+ 0.160 ± 0.043	+ 0.009 ± 0.011	− 2.653	< 0.01
Walking/trailing	+ 0.197 ± 0.035	+ 0.201 ± 0.037	+ 0.002 ± 0.023	− 5.218	< 0.001
Running/leading	− 0.023 ± 0.023	+ 0.009 ± 0.026	+ 0.030 ± 0.011	− 9.727	< 0.001
Running/trailing	− 0.039 ± 0.028	− 0.006 ± 0.024	+ 0.035 ± 0.015	− 9.956	< 0.001
Step length					
Walking/leading	+ 0.145 ± 0.037	+ 0.180 ± 0.053	+ 0.032 ± 0.024	− 13.351	< 0.001
Walking/trailing	+ 0.197 ± 0.035	+ 0.227 ± 0.071	+ 0.025 ± 0.046	− 7.572	< 0.001
Running/leading	− 0.023 ± 0.023	+ 0.022 ± 0.032	+ 0.048 ± 0.016	− 32.762	< 0.001
Running/trailing	− 0.039 ± 0.028	+ 0.010 ± 0.053	+ 0.051 ± 0.027	− 16.022	< 0.001
Peak toe height					
Walking/leading	+ 0.145 ± 0.037	+ 0.079 ± 0.035	− 0.067 ± 0.018	52.531	< 0.001
Walking/trailing	+ 0.197 ± 0.035	+ 0.003 ± 0.030	− **0.200 ± 0.026**	118.662	< 0.001
Running/leading	− 0.023 ± 0.023	− 0.080 ± 0.022	− 0.054 ± 0.012	71.632	< 0.001
Running/trailing	− 0.039 ± 0.028	− 0.064 ± 0.020	− 0.026 ± 0.020	19.316	< 0.001

Analysis of inter-group differences in conjunction with Fig. 4B, with parameters according to Eq. 2. B_G1_ and β_G2_ list bootstrapped mean and sd of the inter-group differences success rate of the null and factor models, respectively, for any gait × leg combination. Δβ_12_, t and p list the corresponding mean and sd of pairwise differences between null and factor models, t-statistic and *p* value. While all parameters result in significant changes, only the introduction of peak toe height results in a major reduction in the inter-group differences (highlighted in bold). Note that success rates (*β*) are given in the range [0, 1], thus corresponding to percentages 0 to 100%

As listed in Table 2 the inter-group difference Δβ_12_ was significantly different from zero for all models, which we attribute to the large number of bootstrap samples. The inter-group factor β_G1_ of the null model (Table 2, first column) showed a 19.7 percent point (trailing foot) and 14.5 percent point (leading foot) inter-group difference in success rate in walking trials. This shows that success rate gradually improved during walking (Fig. 3) and that this improvement differed between cohorts (Fig. 3B). In contrast, β_G1_ amounted to less than 4% in running trials.

Of the three parameters incorporated in the full model, only the *peak toe height* of the trailing foot in the walking condition stood out with a particularly strong effect on the inter-group difference. With Δβ_12_ reaching 20 percent points, the introduction of peak toe height reduced the group factor β_G2_ close to zero (0.003 ± 0.030 percent points). Similarly, factor peak toe height affected Δβ_12_ more consistently than the other two factors, as indicated by the smaller range of variation and number of outliers in Fig. 4B.

**Fig. 5:**
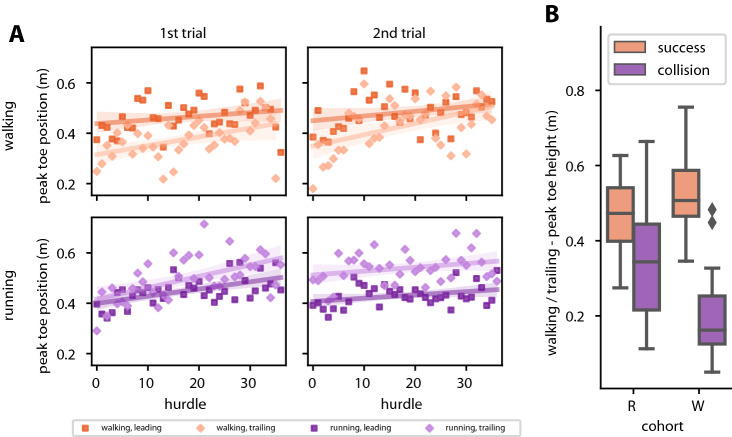
**A** Change of peak toe height over time. Panels separate data according to gait (rows) and trial number (columns). Darker colors show data of the leading foot; lighter colors of the trailing foot. Insufficient trailing toe height played a significant role in unsuccessful traversals. Participants in cohort R quickly adapted their toe height during the subsequent walking trial. Note that the two corresponding trials per cohort are arranged diagonally, so as to emphasize differences between the 1st and 2nd trial measures of the same gaits. **B** Peak toe height differs significantly only in the trailing foot in walking trials. Box plots comprise all trajectories for this gait/foot combination (*n*, from left to right = 113/87; 146/50). There is a significant interaction between this parameter and participant ID, i.e., it varies across trials of each individual. Boxplots show medians, 1st and 3rd quartiles, while whiskers show the minimum and maximum values, with outliers marked individually whenever exceeding 1.5 times the interquartile range beyond the box

Figure 5A illustrates how the parameter peak toe height changed over time as participants gradually improved in walking trials. Especially the peak trailing toe height increased significantly over time, this was true in both walking and in first running trials (Spearman, all *p* < 0.01) but not in the second running trial (Spearman, *p* = 0.248) directly corresponding to the success rates in Fig. 3B. The peak leading toe height only increased significantly in the first running trial (Spearman, *p* < 0.01).

When testing for differences in trailing peak toe height depending on success between cohorts in walking trials (Fig. 5B), we found significant differences between successful and colliding traversals in both cohorts.

This difference was 0.31 m for cohort *W* vs. 0.14 m for cohort R (Mann–Whitney *U*; cohort *W*, *n*_success_ = 113, *n*_collision_ = 87, *U* = 1048, *p* < 0.001; cohort R, *n*_success_ = 146, *n*_collision_ = 50, *U* = 2049, *p* < 0.001). Note that this includes all traversals, not averaged per participant, as the effect was lost in the averaging process. We conclude that the difference is found in the extremes of each participant, not their averages.

Similarly, a small but significant increase was found in the intercept of the peak toe height of the trailing foot between the two cohorts. Cohort R had an initial toe height of 34.41 ± 33.60 cm, compared to 28.89 ± 42.73 cm in cohort W (*t* Test: *Δ*_height_ = 5.62 cm, *t* = − 2.11, *p* < 0.05, *n* = 430/410). Again this difference is present only when not averaging per participant. No difference in performance was observed in the peak toe height of the leading foot (*t* Test: *Δ*_height_ = 3.05 cm, *t* = − 1.318, *p* = 0.187, *n* = 432/412) nor in the slope of leading or trailing foot peak toe height.

We conclude that lacking toe height of the trailing foot is the main factor underlying the difference in improvement between cohorts and, therefore, in the asymmetry of the transfer across gaits.

## Discussion

We showed that participants were able to transfer learned adjustments of trailing foot height between gaits (Fig. 3A), but this transfer depended on gait order (Fig. 3B). The group that started out with running achieved higher success rates in a subsequent walking trial compared to the group that started out with walking. Toe height of the trailing foot was identified as the key parameter for improving the success rate of obstacle traversal (Figs. 4B, 5).

### Overall performance and potential effects of VR

Success rates varied across participants, ranging from 7 to 100%, with an average success rate of 70% in exp. 2. Compared to previous studies, our success rates were considerably lower than the 98–99% reported for stationary, physical obstacles (Heijnen et al. 2012; Muir et al. 2015) and the 98–99% for stationary, virtual obstacles (Binaee and Diaz 2019). Averaged across the entire experiment and both cohorts, we did not observe higher collision rates in trailing foot compared to leading foot trajectories. This is in contrast to Heijnen et al. (2014), where mean collision rates were higher for the trailing foot.

One reason for lower success rates may be attributed to the fact that we used higher hurdles compared to the ~ 20 cm obstacle height used in other studies (Heijnen et al. 2014; Heijnen and Rietdyk 2018; Patla and Greig 2006) reducing the success rates by virtue of difficulty. A similar effect was found by Coolen et al. (2020), where most collisions were attributed to higher obstacles. Another factor affecting success rate is the mode of projection of the virtual obstacles. For example, our success rates were considerably higher than those reported by LoJacono et al. (2018) who presented virtual obstacles in a third-person perspective on a screen in front of the participant. In contrast, like Binaee and Diaz (2019), we provided an immersive first-person projection, allowing the participants to perceive the obstacle in the correct position relative to their body. In our setup, obstacles were not visible during traversal, though their relative location was marked by lateral, vertical poles. Restricting obstacle visibility is known to reduce success rates by up to 50% (LoJacono et al. 2018; Mohagheghi et al. 2004; Patla 1998), which is substantially less than in our study. Not being able to see the obstacle at the moment of traversal may not be critical for successful traversal, as motion planning occurs two steps ahead of the hurdle (Binaee and Diaz 2019; Diaz et al. 2018; Matthis and Fajen 2013; Patla and Vickers 2003). Moreover, Heijnen et al. (2014) showed that it is possible to traverse obstacles from memory that have been observed, but were removed before traversal. In our setup, the distance at which the obstacle left the screen area depended on participant height, obstacle height and observer position on the treadmill. The upper boundary of this distance would have occurred for the lowest body size (1.6 m) and obstacle height (0.1 m), amounting to 1.7 m before the traversal. With a set treadmill speed, an assumed central position on the platform and a given height of the participant, the number of steps after the obstacle left the field of view depended on step length only. Assuming at least 1 m step length for runners and 0.6 m for walkers, this should have affected walking results more than running results. It should be noted, that we did not find a significant correlation of success rates and participant height, nor was lift-off distance or step length critical in explaining the inter-group difference. One participant was not able to perform the task successfully. Similar inabilities were found by Coolen et al. (2020) in an AR obstacle paradigm. There, the majority of collisions were attributed to three participants only. This suggests a form of AR/VR “blindness”, a potential subject for future experiments.

We conclude that participants were affected only little by not being able to observe the obstacle during traversal. The success rates observed in our experiment are in line with those reported in previous studies, with the spatial visualisation limit of our VR setup and increased obstacle height being the most likely factors for reduced success rates.

More generally, the question whether experiments in artificial environments with treadmill and VR can be valid substitutes for natural over-ground locomotion has been answered controversially. Having tactile feedback is necessary to judge trajectory heights accurately (Heijnen et al. 2012; Heijnen and Rietdyk 2018), but a purely virtual environment cannot provide this. While the equivalence of treadmill vs. overground running has been critized in the past (e.g., Nelson et al. 1972; Nigg et al. 1995), several studies have found treadmills to be a valid surrogate paradigm for locomotion research (Bassett et al. 1985; Riley et al. 2007; van Ingen Schenau 1980). It is commonly assumed that participants behave the same in a virtual reality setup (Binaee and Diaz 2019; LoJacono et al. 2018). As yet, the lack of stereo-vision (Hayhoe et al. 2009), lacking visual information during the last steps (Binaee and Diaz 2019) and the awareness of an unnatural situation could have affected participant behavior.

### Improvement with experience and transfer between gaits

Participants in running trials started at high success rates and gained 12–16 percent points over the course of the experiment. In contrast, the increase in success rate was larger in walking trials, with a gain of 29–33 percent points. Following a running trial, the learning curve for walking started at significantly higher average success rates.

Toe trajectories of our participants for walking and running were very similar (Fig. 2E), consistent with the finding that "toe trajectories are strikingly well conserved" (Lam and Dietz 2004) over a wide range of conditions such as forward vs. backwards walking (Grasso et al. 1998) or upright walking vs. crouching (Grasso et al. 2000). Lam and Dietz (2004) showed that it was possible to train participants to control the height of foot trajectories over obstacles within a very narrow range and found that this spatial (height) constraint transferred well to obstacle traversals on a downhill slope or with added a weight at the ankles (Lam and Dietz 2004). It seems reasonable to assume that, like the gaits themselves (Geyer et al. 2006; Hildebrand 2013), spatial motor adjustments for obstacle traversal are subject to a shared control mechanism.

From a computational viewpoint, invariance of movement parameters of a redundant motor system indicates control by the central nervous system (Bernshteĭn, 1967). Following this rationale, spatial invariance of motor adjustments during obstacle traversal indicates that the endpoint trajectory of the foot—specifically its height and lift-off position—should be controlled by appropriate adjustment of joint kinematics and dynamics (Bosco and Poppele 2001). Accordingly, the spatial foot trajectory should vary little, even when learned spatial adaptations are transferred from one experimental situation to the next, for example, to the opposite leg (van Hedel et al. 2002), between modalities (Erni and Dietz 2001) or from VR to the real world (LoJacono et al. 2018) or from running to walking (this study). Overall, this suggests that the spatial information needed to cross obstacles is stored independently of the actual motor programs and thus should be free to transfer over a wide range of conditions, including between gaits. This is consistent with the view of a shared control mechanism for walking and running (Cappellini et al. 2006; Geyer et al. 2006; Hildebrand 2013).

Following this idea, our cohort R was able to adjust the parametrisation of a common control system responsible for obstacle traversal in response to encountering obstacles in the running trial, infer information necessary to successfully cross the obstacles and use that knowledge to quickly adjust in the subsequent walking trials leading to the high initial success rates. This information may have been gathered visually (Patla and Vickers 1997), proprioceptively, and in conjunction with auditory feedback (Erni and Dietz 2001).As we can rule out insufficient peak CoM height (Fig. 4A) and step positioning (Fig. 4B), we conclude that cohort R has learned sufficient toe height in the running trial which lead to improved performance in the subsequent walking trial. Indeed, our statistical modelling results attest peak toe height to be the best explanation for a difference between the two cohorts in the walking trials. Low success rates in the walking trial can then be attributed to lacking height of the trailing foot, consistent with studies by Heijnen et al. (2014) and Muir et al. (2015). Indeed, trailing toe height increased over the course of both walking and running trials. In contrast, other studies found a decrease in toe height over time (Heijnen and Rietdyk 2018), though with much longer trials and about fourfold obstacle numbers. Assuming that gait kinematics are the result of minimizing energy expenditure at maximum success (Heijnen et al. 2012; Loeb 2012), the persistent increase in toe height in our study suggests that the learning process was not finalized after 36 traversals. This is supported by the fact that peak toe height variability was still high at the end of the first and moderately high at the end of the second trials, especially in walking (Fig. 5A).

### Inter-gait learning transfer is not symmetrical

Our original expectation was that inter-gait learning transfer—if any was to be detected—would be bi-directional. This was clearly not the case. Instead, transfer occurred from running to walking, but not the other way round. After finishing the walking trial, cohort W started their running trial at the same success rate as cohort R, but did improve over the course of the trial. We see this as evidence that there was no transfer from walking to running in cohort W, as we would have expected to see an improvement in initial success rate over cohort R. Therefore, we conclude that motor adjustments during obstacle traversal is not strictly gait-dependent, but subject to an asymmetry in the transfer between gaits. A potential explanation for this asymmetry could be linked to the fact that the obstacle course was exactly the same for running and walking trials, leaving the possibility that hurdles were more difficult to be crossed during walking that during running. In other words, difficulty might have been too low to observe a significant difference between the overall success rates of running trials. Indeed, both cohorts performed equally well in the running trials, irrespective of previous experience during walking. However, the mere presence of an increase in success rate during a preceding walking trial means that participants did improve during walking, but that this improvement failed to affect the onset of the subsequent learning curve of the running trial. Future experiments will need to test whether a change in trial difficulty during running may reveal learning transfer from walking to running, too.

## Supplementary Information

Below is the link to the electronic supplementary material.

Supplementary file1 (PDF 38 KB)

Supplementary file2 (PDF 37 KB)

## References

[CR1] Alexander RM (1984). Walking and running: legs and leg movements are subtly adapted to minimize the energy costs of locomotion. Am Sci.

[CR2] Alexander RM (2013). Principles of Animal Locomotion.

[CR3] Bassett JD, Giese MD, Nagle FJ, Ward A, Raab DM, Balke B (1985). Aerobic requirements of overground versus treadmill running. Med Sci Sports Exerc.

[CR4] Bernshteĭn NA (1967). The Co-ordination and Regulation of Movements.

[CR5] Binaee K, Diaz GJ (2019). Assessment of an augmented reality apparatus for the study of visually guided walking and obstacle crossing. Behav Res Methods.

[CR6] Bosco G, Poppele RE (2001). Proprioception from a spinocerebellar perspective. Physiol Rev.

[CR7] Cappellini G, Ivanenko YP, Poppele RE, Lacquaniti F (2006). Motor patterns in human walking and running. J Neurophysiol.

[CR8] Chou L-S, Draganich LF (1998). Placing the trailing foot closer to an obstacle reduces flexion of the hip, knee, and ankle to increase the risk of tripping. J Biomech.

[CR9] Coolen B, Beek PJ, Geerse DJ, Roerdink M (2020). Avoiding 3D obstacles in mixed reality: does it differ from negotiating real obstacles?. Sensors.

[CR10] Diaz GJ, Parade MS, Barton SL, Fajen BR (2018). The pickup of visual information about size and location during approach to an obstacle. PLoS ONE.

[CR11] Erni T, Dietz V (2001). Obstacle avoidance during human walking: learning rate and cross-modal transfer. J Physiol.

[CR12] Geyer H, Seyfarth A, Blickhan R (2006). Compliant leg behaviour explains basic dynamics of walking and running. Proc r Soc B Biol Sci.

[CR13] Grasso R, Bianchi L, Lacquaniti F (1998). Motor patterns for human gait: backward versus forward locomotion. J Neurophysiol.

[CR14] Grasso R, Zago M, Lacquaniti F (2000). Interactions between posture and locomotion: motor patterns in humans walking with bent posture versus erect posture. J Neurophysiol.

[CR15] Hayhoe M, Gillam B, Chajka K, Vecellio E (2009). The role of binocular vision in walking. Vis Neurosci.

[CR16] Heijnen MJH, Rietdyk S (2018). Failures in adaptive locomotion: trial-and-error exploration to determine adequate foot elevation over obstacles. Exp Brain Res.

[CR17] Heijnen MJH, Muir BC, Rietdyk S (2012). Factors leading to obstacle contact during adaptive locomotion. Exp Brain Res.

[CR18] Heijnen MJH, Romine NL, Stumpf DM, Rietdyk S (2014). Memory-guided obstacle crossing: more failures were observed for the trail limb versus lead limb. Exp Brain Res.

[CR19] Hildebrand M (2013). Chapter 3.

[CR20] Lam T, Dietz V (2004). Transfer of motor performance in an obstacle avoidance task to different walking conditions. J Neurophysiol.

[CR21] Loeb GE (2012). Optimal isn’t good enough. Biol Cybern.

[CR22] LoJacono CT, MacPherson RP, Kuznetsov NA, Raisbeck LD, Ross SE, Rhea CK (2018). Obstacle crossing in a virtual environment transfers to a real environment. J Mot Learn Dev.

[CR23] Matthis JS, Fajen BR (2013). Humans exploit the biomechanics of bipedal gait during visually guided walking over complex terrain. Proc r Soc B Biol Sci.

[CR24] Mauroy G, Schepens B, Willems PA (2013). The mechanics of running while approaching and jumping over an obstacle. Eur J Appl Physiol.

[CR25] Mohagheghi AA, Moraes R, Patla AE (2004). The effects of distant and on-line visual information on the control of approach phase and step over an obstacle during locomotion. Exp Brain Res.

[CR26] Mori S, Sakamoto T, Ohta Y, Takakusaki K, Matsuyama K (1989). Site-specific postural and locomotor changes evoked in awake, freely moving intact cats by stimulating the brainstem. Brain Res.

[CR27] Muir BC, Haddad JM, Heijnen MJH, Rietdyk S (2015). Proactive gait strategies to mitigate risk of obstacle contact are more prevalent with advancing age. Gait Posture.

[CR28] Muir BC, Bodratti LA, Morris CE, Haddad JM, van Emmerik REA, Rietdyk S (2020). Gait characteristics during inadvertent obstacle contacts in young, middle-aged and older adults. Gait Posture.

[CR29] Nelson RC, Dillman CJ, Lagasse P, Bickett P (1972). Biomechanics of overground versus treadmill running. Med Sci Sports.

[CR30] Nigg BM, De Boer RW, Fisher V (1995). A kinematic comparison of overground and treadmill running. Med Sci Sports Exerc.

[CR31] Patla AE (1998). How is human gait controlled by vision. Ecol Psychol.

[CR32] Patla AE, Greig M (2006). Any way you look at it, successful obstacle negotiation needs visually guided on-line foot placement regulation during the approach phase. Neurosci Lett.

[CR33] Patla AE, Rietdyk S (1993). Visual control of limb trajectory over obstacles during locomotion: effect of obstacle height and width. Gait Posture.

[CR34] Patla AE, Vickers JN (1997). Where and when do we look as we approach and step over an obstacle in the travel path?. NeuroReport.

[CR35] Patla AE, Vickers JN (2003). How far ahead do we look when required to step on specific locations in the travel path during locomotion?. Exp Brain Res.

[CR36] Patla AE, Prentice SD, Robinson C, Neufeld J (1991). Visual control of locomotion: strategies for changing direction and for going over obstacles. J Exp Psychol Hum Percept Perform.

[CR37] Patla AE, Rietdyk S, Martin C, Prentice S (1996). Locomotor patterns of the leading and the trailing limbs as solid and fragile obstacles are stepped over: some insights into the role of vision during locomotion. J Mot Behav.

[CR38] Rietdyk S, Rhea CK (2006). Control of adaptive locomotion: effect of visual obstruction and visual cues in the environment. Exp Brain Res.

[CR39] Riley PO, Paolini G, Della Croce U, Paylo KW, Kerrigan DC (2007). A kinematic and kinetic comparison of overground and treadmill walking in healthy subjects. Gait Posture.

[CR40] Sparrow WA, Shinkfield AJ, Chow S, Begg RK (1996). Characteristics of gait in stepping over obstacles. Hum Mov Sci.

[CR41] Tredinnick, R., Boettcher, B., Smith, S., Solovy, S., Ponto, K., 2017. Uni-CAVE: A Unity3D Plugin for Non-head Mounted VR Display Systems, in: Virtual Reality (VR), 2017 IEEE. IEEE, p. In Print.

[CR42] van Ingen Schenau GJ (1980). Some fundamental aspects of the biomechanics of overground versus treadmill locomotion. Med Sci Sports Exerc.

[CR43] van Hedel HJA, Biedermann M, Erni T, Dietz V (2002). Obstacle avoidance during human walking: transfer of motor skill from one leg to the other. J Physiol.

[CR44] Yao R, Heath T, Davies A, Forsyth T, Mitchell N, Hoberman P (2014). Oculus vr best practices guide. Oculus VR.

