# Supplementary figures and images for: When running is easier than walking: effects of experience and gait on human obstacle traversal in virtual reality
Source: Exp Brain Res. 2022 Sep 17;240(10):2701–14. doi: 10.1007/s00221-022-06443-2 (PMC9510118; doi:10.1007/s00221-022-06443-2)

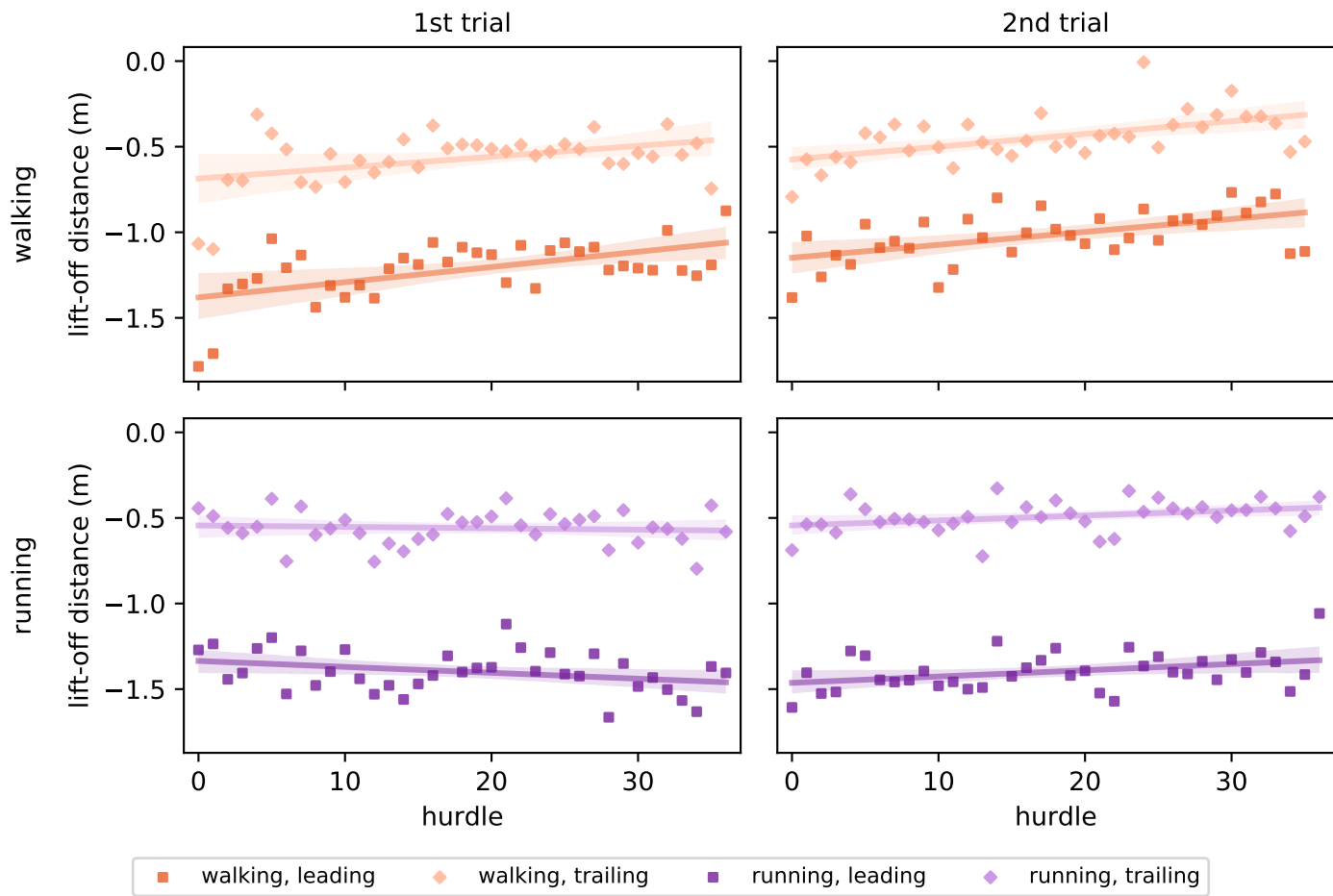

Supplement: Supplementary file 1 — Supplementary file1 (PDF 38 KB) [file 221_2022_6443_MOESM1_ESM.pdf]

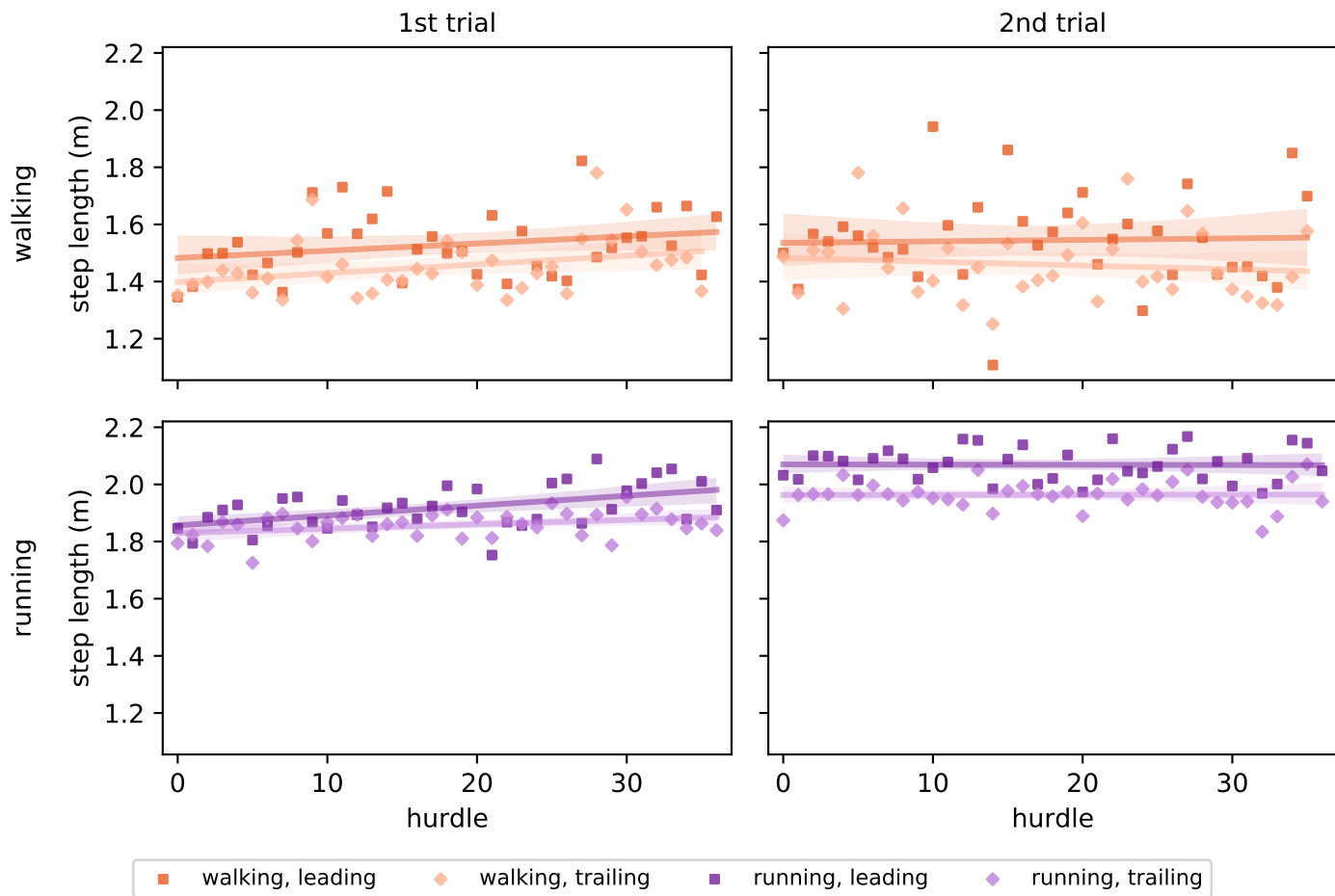

Supplement: Supplementary file 2 — Supplementary file2 (PDF 37 KB) [file 221_2022_6443_MOESM2_ESM.pdf]
